# Do Trait Emotional Intelligence and Dispositional Mindfulness Have a Complementary Effect on the Children’s and Adolescents’ Emotional States?

**DOI:** 10.3389/fpsyg.2019.02817

**Published:** 2019-12-20

**Authors:** Jose M. Mestre, Jorge Turanzas, Maria García-Gómez, Joan Guerra, Jose R. Cordon, Gabriel G. De La Torre, Victor M. Lopez-Ramos

**Affiliations:** ^1^Instituto Universitario para el Desarrollo Social Sostenible (INDESS), Universidad de Cádiz, Cádiz, Spain; ^2^Department of Psychology, Universidad de Cádiz, Cádiz, Spain; ^3^Universidad Nacional de Educación a Distancia (UNED), Algeciras Centre, Cádiz, Spain; ^4^Department of Psychobiology, Universidad de Murcia, Murcia, Spain; ^5^Department of Psychology and Anthropology, University of Extremadura, Cáceres, Spain; ^6^Instituto de Investigación e Innovación en Ciencias Biomédicas de la Provincia de Cádiz (INiBICA), Universidad de Cádiz, Cádiz, Spain

**Keywords:** trait emotional intelligence, ability emotional intelligence, dispositional mindfulness, emotional Intelligence, adolescents

## Abstract

Mindfulness is both a non-judgmental and present-centered awareness, which has been applied to reduce negative emotions. On the other hand, Trait Emotional Intelligence (TEI) is the way of how good people perceive their emotional intelligence abilities (perceiving, expressing, understanding, and regulating emotions), which are involved in people’s social functioning. This empirical study was designed to analyze whether dispositional mindfulness (DM) and TEI have a potential combined role for children and adolescent’s emotional states. In a sample of primary school students (*N* = 318), age ranged from 8 to 16 years old (*M* = 11.25, *SD* = 2.20), participants filled a TEI measure (ESCQ, Emotional skills and competence questionnaire) and two measures of DM (CAMM, Child and Adolescent Mindfulness Measure and AFQ-Y, Avoidance and Fusion Questionnaire for Youth). Measures selected included: PANAS (Positive affect and negative affect schedule), White Bear Suppression Inventory (a thought suppression inventory), and STAIC (State-Trait Anxiety for Children). Findings pointed out that TEI measures (labeling and expression, understanding, and managing emotions) were positively and significantly related to positive emotional states (especially, positive affect and balance) and negatively with a lower association with state anxiety. However, DM measures were both negatively and strongly associated with negative emotional states (thought suppression, negative affect, and anxiety). Conclusions indicate that a combined effect of both TEI skills and DM based interventions would be more complete than each one separately for better social functioning of children and teenagers.

## Introduction

The mental and emotional health of both primary and secondary students is a growing concern. There is a relationship between the low-level adaptive-emotional states and serious outcomes regarding both academic (Jayalakshmi and Magdalin, 2015; Frajo-Apor et al., 2016) and social adaptation to school (Mestre et al., 2006).

When a person reaches adolescence, social-emotional development begins to have an important impact with several relevant outcomes in their lives. For example, this may have an effect on having an adequate social identity (Van Tilburg and Igou, 2011), good social functioning with peers (Lopes et al., 2012), better possibilities of school adaptation (Nathanson et al., 2016), and better mental health and well-being (Afzal et al., 2016). Therefore, everything that favors an adjustment in the adolescents’ affective sphere is of interest to be included in their training processes (Gross and John, 2003). According to the accumulated empirical evidence, two factors seem to have positive impacts on the emotional states at the beginning of adolescence: emotional intelligence (f. i., Mavroveli et al., 2008; Laborde et al., 2014; Perera, 2016) and dispositional mindfulness (f. i., Cullen, 2011; Hinterman et al., 2012; MacBeth and Gumley, 2012).

In the case of EI, two ways of conceptualizing it seem to coexist. On one hand, *Ability Emotional Intelligence* (AEI), which considers EI as a set of cognitive abilities (perceiving, using, understanding, and managing) for making decisions based on hot information processing — it refers to all information that has a vital meaning for people (Mayer et al., 2016) for adequate personal and social functioning (Peña-Sarrionandia et al., 2015). However, some authors have considered not including “the emotional using” branch in the EI framework because they believe it should be considered as part of the other three branches (Mestre et al., 2016). On the other hand, *Trait Emotional Intelligence* (TEI) has been defined as a set of emotional self-perceptions placed at the lower standards of personality hierarchy (Petrides et al., 2016). There are also two different approaches to measure EI. While AEI advocates the exclusive use of performance instruments (Brackett and Salovey, 2006) and/or emotional-situation judgment tests (MacCann and Roberts, 2008), like any other type of intelligence, the TEI just uses self-reported instruments, like the TEIQue (Trait Emotional Intelligence Questionnaire, Laborde et al., 2016).

However, when non-adult samples are used to measure emotional intelligent behaviors, investigations have shown stronger relationships with both negative (see Windingstad et al., 2011) and positive (see Mavroveli et al., 2008) emotional states with TEI measures than with AEI ones. Nonetheless, we should admit that the AEI framework has shown advantages (Fiori, 2009), but the theoretical AEI framework might be better explained using the Cattell-Horn-Carroll mental abilities model and emotional appraisal theory (Roberts et al., 2016) since measures of AEI only have assessed a part of the emotional intelligent behavior, the emotional conscious part (Fiori, 2009; Fiori and Antonakis, 2011). Hence, AEI measures still lack information about how extent emotions, AEI measures, and intelligence are related (Roberts et al., 2006; Maul, 2012). Especially for under-17-year-old samples, it would a possibility to build a TEI instrument according to the AEI theoretical foundations (Billings et al., 2014; Davis et al., 2015). Therefore, we used a self-report of AEI (Faria et al., 2006) that encompasses TEI principles to assess emotional intelligent behavior (Azghandi et al., 2007) to study its relationships with both positive and negative emotional outcomes.

Regarding Mindfulness tradition and theories among adolescents, this concept used to be defined as a quality of consciousness (Brown et al., 2009), which implies drawing attention to the experiences occurring in the present moment and developing a non-judgmental accepting awareness of moment-by-moment experience (Kabat-Zinn, 2003). However, *dispositional mindfulness* (DM) has been defined as a trait in which individuals differ in how they accept and live with commitment (Goodall et al., 2012). Hence, DM might be a predisposition or trait for living in a mindfulness way (Petrides et al., 2017; Turanzas et al., 2018).

A systematic review by Tomlinson et al. (2017) reported how DM was related to the psychological health of people. The authors reviewed non-interventional and quantitative DM’s articles in non-clinical samples. According to their review, DM was negatively related to non-adaptive emotions (such as anxiety or depression symptoms), and positively linked to adaptive cognitive strategies of emotional regulation processes (such as reappraisal and acceptance) and positive emotions (f. i., joyful) (Tomlinson et al., 2017). Similar findings have been reported in samples of adolescents, in which DM was positively related to subjective well-being (Brown et al., 2009), but mostly DM showed negative relationships with some criteria that indicated a certain level of protection, such as lower levels of dysphoric mood and better tolerance to the effects of stress (Ciesla et al., 2012), lower social anxiety (Hambour et al., 2018), and even in gifted adolescents lower levels of depression, anxiety, and negative emotions have been found (Turanzas et al., 2018).

However, there are few and incomplete studies which have included both EI and DM. Most of these studies have used either DM (from EI-experienced authors) or EI as a mediating variable (conversely, from DM-experienced authors). Hence, there is not enough evidence yet to establish moderating and/or mediating factors between DM and EI regarding positive or negative affective criteria. For example, Teal et al. (2018) studied the influence of EI and DM in the well-being of school programs in a sample of 294 male adolescents. Authors hypothesized that EI mediated the relationship between DM and wellbeing (measured using subjective happiness and psychological stress). Findings supported their previous ideas, using multiple mediation models, where different dimensions of EI (measured with SUEIT, Swinburne University Emotional Intelligence Test, Palmer and Stough, 2001) were significantly and positively related to DM. Besides, EI partially mediated the relationship between DM and subjective happiness. However, the authors were not persuasive enough in explaining the reason why they considered DM as a criterion instead of a predictor. Another concern was that the authors did not include female participants so we cannot know to what extent gender roles affected (a potential qualitative moderating factor). Another example, Park and Dhandra (2017a), studied an Indian sample with 319 participants to ascertain whether TEI (using the WLEIS, Shi and Wang, 2007) might moderate the relationship between DM and the impulsive buying tendency. The authors checked this mediator role of TEI and showed that DM was positively related to all components of the TEI measure. Therefore, it is unclear how DM and EI work together, or separately, or under mediating or moderating factors.

Consequently, both previous DM-EI antecedents, mentioned above, pointed to DM as a predictive factor without considering TEI as a predictive variable too, and without enough theoretical explanations of why TEI had to be viewed as mediator. Some interesting reviews about AEI (see Fernandez-Berrocal and Extremera, 2016) or TEI (see Petrides et al., 2016) have highlighted EI as a proven moderator of health outcomes rather than a mediator. However, they have also pointed out that EI’s role as a mediator is still at an early stage.

Following Kazdin (2007), a moderator variable affects the direction and strength between predictors and criteria. However, a mediator variable counts as “a milestone” for the relationship between predictors and criteria. While mediators explain how external variables take on internal psychological significance, moderators highlight when and/or how much the effects will affect a criterion. Hence, how should the DM and/or EI role be explained as a mediator or as a moderator? Some recent studies may have clarified this question.

For example, Miao et al. (2018) conducted a meta-analytic revision of 17 eligible studies to examine in greater depth the potential relationship between EI (no AEI measures were included in the review, just TEI measures) and trait mindfulness (or DM). They concluded that it was not possible to identify all potential moderators in this EI-DM relationship. However, they pointed out that future research should examine “the relationships among EI, mindfulness, and various other outcome variables need further examination and replication” (Miao et al., 2018, p. 106). However, in their review, they state that the DM and the TEI can be both mediators and moderators.

We have also found some more related antecedents such as emotion regulation (ER), as a mediator, where DM was related to some emotional criteria. For example, in a sample of 572 adolescents, it was found that the ER difficulties mediated the relationship between DM and anxious attachment (Pepping et al., 2016). Ciesla et al. (2012) also described a mediating role of ER between DM and psychological health. However, others consider DM has an assisting role with ER (Hambour et al., 2018).

In our opinion, we believe that both DM and EI (including ER, TEI or AEI) require more attention and research on whether some of them have a potential mediating or moderating role in the relationships sought, with interesting criteria for both constructs, such as self-personal (self-cognitive/emotional regulatory processes) and/or social functioning (social anxiety, for example). Especially in heterogeneous samples, like adolescents (without scholar filters yet), it is necessary to explore previously what type of relationships EI, in our case TEI, and DM have with criteria of positive and negative affectivity before considering different models of mediation. The need to study both DM and self-regulatory processes with more attention were already pointed out by Masicampo and Baumeister (2007) since we are still in the initial stages of how DM or TEI are related to adolescents’ affective sphere.

The main purpose of this cross-sectional study, in a sample of Spanish children and adolescents, was to observe the potential significant relations of TEI and DM may have with various criteria of the affective sphere, anxiety trait vs. state, psychological flexibility and the suppression of thought. We also explored the potential mediating effects between TEI and DM with both positive and negative affect of children and adolescents. We hypothesized that while TEI is positively associated with the positive emotional states and negatively with thought suppression, DM will negatively relate to both negative affect and non-adaptive states.

## Materials and Methods

### Participants and Procedure

The sample comprised 318 Spanish children and adolescents who were selected by quota sampling from various primary and secondary schools in Cadiz (South of Spain). The average age was 11.25 (range = 8–16, *SD* = 2.20). Subjects were nearly equally divided by gender (49.1% female). Participants completed questionnaires during class time in a single session. Participation was anonymous and voluntary, and data collection followed the ethical guidelines applicable to people under 18. Before completing the questionnaire, participants also presented parental authorization. The ethical recommendations led to 21 participants being excluded from the sample for not providing informed consent from their parents.

### Instruments

#### TEI (Trait Emotional Intelligence)

The *Spanish version of the Emotional Skills and Competences Questionnaire* (ESCQ, Takšić, 2009) was used to measure emotional competence. This self-report measure was developed using the Mayer and Salovey (1997) model of Emotional Intelligence (EI, perceiving, using, understanding, and managing emotions). Participants had to rate the items on 5-point scales (1-never, 2-seldom, 3- occasionally, 4-usually, 5-always). The short version consists of 45 items combined into three subscales: *Perceiving and understanding emotions*, which has 15 items (e.g., “When I see how someone feels, I usually know what has happened to him”); *Expressing and Labeling emotions* scale, 14 items (e.g., “I am able to express my emotions well”), and *Managing and Regulating emotions* scale, 16 items (e.g., “When I am in a good mood, every problem seems soluble”). The questionnaire was translated into more than ten languages and shows good reliability and constructive validity (Faria et al., 2006). We used an average score (from 1 to 5) for each ESCQ’s scale.

#### Dispositional Mindfulness

To assess DM, we used the *Child and Adolescent Mindfulness Measure* (CAMM; Greco et al., 2011). The CAMM consist of 10 items, responded to on a 5-point Likert scale, ranging from 0 (never true) to 4 (always true). Lower scores would indicate a disposition for having mindful skills in everyday life. This measure is based on the Kentucky Inventory of Mindfulness Skills (KIMS) (Baer et al., 2004), which assesses acting with awareness of the present moment and accepting without judgment. For this study, we used an 8-item CAMM’s Spanish adaptation of Turanzas (2013), which has shown good psychometric properties (Cronbach alpha = 0.82). Turanzas’s CAMM version erased two items (#5 and #10) due to among children produced biased interpretations and misunderstandings. Similar issues have been found in non-English versions (for instance, Italian see Saggino et al., 2017). In this study, we computed this measure as the averaged total score (from 0 to 4). Since items were written in a negative sense, we also recoded the scoring for an easier interpretation. Hence, higher scores indicated higher DM.

## Criteria

### Cognitive Styles Related to Emotional Functioning

#### Psychological Inflexibility: Cognitive Fusion and Experiential Avoidance

Psychological inflexibility was measured using the Spanish adaptation of Avoidance and Fusion Questionnaire for Youth (Spanish version of AFQ-Y, Valdivia-Salas et al., 2016). Greco et al. (2008) developed the original instrument to measure psychological inflexibility, comprised of two subscales: cognitive fusion and experiential avoidance, in children and adolescents. According to Hayes et al. (2006), *cognitive fusion* refers to how verbal processes interfere in the regulation of behavior, while *experiential avoidance* explains how people react to private of events “even when doing so causes behavioral harm” (p. 7). The AFQ-Y has 17-Likert items ranged from “0” (not at all true) to “4” (very true). Example items include “I must get rid of my worries and fears, so I can have a good life” and “I push away thoughts and feelings that I don’t like.” High scores imply a trend to fuse with own thoughts and feelings. This measure only assesses the negative tendency of cognitive flexibility. Greco et al. (2008) found high Cronbach reliability. For this study average scores were used (from 0 to 4).

#### Thought Suppression and Intrusion

The *White Bear Suppression Inventory* (WBSI, Wegner and Zanakos, 1994). Based on previous ideas (Wegner et al., 1987), this instrument comprises 15 items to evaluate chronic thought suppression tendencies. The respondents are requested to indicate their agreement with statements on a 5-point Likert scale ranging from 1 “strongly disagrees” to 5 “strongly agree.” Thus, the total score ranges from 15 to 75, with higher scores indicating greater tendency to suppress unwanted thoughts. It contains statements such as “There are things I prefer not to think about” or “I always try to put problems out of mind.” The WBSI has demonstrated high internal consistency in Spanish (and Portuguese) samples (Ros et al., 2015). This inventory is an indicator of the frequency individuals have intrusive and ruminative thoughts and has been found to correlate positively with depressive symptoms, anxiety, and obsessive-compulsive behavior (Wegner and Zanakos, 1994). For this study, we used average scores (from 1 to 5). According to Schmidt et al. (2009), two subscales would be used (six items each one) - Suppression and Intrusion thoughts.

### Emotional and Affective Criteria

#### Positive and Negative Affect

*The Positive and Negative Affect Schedule for Children PANAS-C* (Spanish validation is named as “PANASN” by Sandín, 2003). The PANASN was based on the original instrument (Laurent et al., 1999). This is a 30-item measure for children and young adolescents, which assesses Positive affects (PA; e.g., cheerful) and Negative affect (NA; e.g., lonely) using 15 items for each. PANASN also provides a measure of *Balance* (PA – NA). Participants were asked to describe how they felt during the past few weeks on a 5-point Likert scale ranging from 1 “slightly or seldom” to 3 “much or often.” Spanish PANASC (PANASN) has shown appropriate values of internal consistency, as well as convergent and discriminant validity (Sandín, 2003). For this empirical study, an average score of PANASN was used (from 1 to 3).

#### Anxiety State-Trait

The State-Trait Anxiety Inventory for Children (STAIC; Spanish version see Spielberger et al., 1982) is a self-report measure which has been widely used to assess state and trait anxiety of children and adolescents. This questionnaire contains two separate, 20-item self-report rating scales for measuring trait and state anxiety. The participant is asked to rate on a 3-point scale the degree to which they are currently experiencing a particular symptom (e.g., I feel 1-not scared, 2-scared, and 3-very scared). Total anxiety scores for the state-anxiety and trait-anxiety scales are obtained by adding up the scores for the 20 items on each scale. Total scores for situational and baseline questions range separately from 20 to 60, with higher scores denoting higher levels of anxiety. Nonetheless, we did not use average scores for STAIC due to their computation.

### Data Analysis

To describe features of the sample we conducted a simple analysis to report the sample. To find relevant relations among variables, we conducted Pearson correlations. Probably, the study will have signs of collinearity due to it has several variables as subscales of similar theoretical constructs. In order to find significant relationships, we did regression analyses and mediating and moderating studies using the MACRO plugin in SPSS.

## Results

Descriptive data and reliability indices are summarized in Table 1 and correlations between variables are reported in Table 2.

**TABLE 1:** Descriptive values for the empirical study (*N* = 316, 49.1% females).

**Variables**	***MIN***	***MAX***	***M***	***SD***	**α**
Age	8	16	11.25	2.20	−
**Trait Emotional Intelligence-TEI (measured using ESCQ, ranged all from 1 to 6)**					
ESCQ_Total	1	5.98	4.30	0.90	0.95
Perceiving and understanding emotions	1	6	4.33	0.90	0.87
Expressing and Labeling emotions	1	6	4.37	0.89	0.8
Managing and regulating emotions	1	6	4.2	0.84	0.84
**Dispositional Mindfulness (measured using Spanish CAMM-8, ranged from 0 to 4)**					
CAMM (DF children and adolescents)	0.13	4	2.81	0.80	0.79
**Cognitive Style Criteria (AFQ-Y 16, ranged from 0 to 4, and WBSI ranged from 1 to 5)**					
AFQ Total	0	3.53	1.47	0.72	0.82
Fusion cognition	0	3.38	1.07	0.70	0.65
Experiential Avoidance	0	4	1.87	0.89	0.76
WBSI (White Bear)	1	4.93	3.11	0.88	0.88
Suppression of thoughts	1	5	3.16	0.99	0.76
Intrusion of thoughts	1	5	3.03	0.93	0.73
**Affective Criteria (PANASN ranged from 1 to 3) and Spanish STAI-C (from 20 to 60)**					
Positive-affect	1	3	2.35	0.37	0.72
Negative-affect	1	2.6	1.61	0.39	0.76
Balance	−0.80	2	0.74	0.58	-
Anxiety state	20	55	29.97	6.41	0.82
Anxiety trait	20	54	3.77	7.19	0.85

*Cronbach reliabilities for the study are also reported.*

**TABLE 2:** Intercorrelations of empirical study’s variables.

**Variables**	**1**	**2**	**3**	**4**	**5**	**6**	**7**	**8**	**9**	**10**	**11**	**12**	**13**	**14**	**15**	**16**	**17**
1 Age	−																
2 Gender	–0.02	−															
3 ESCQ	0.04	0.11*	−														
4 PU	0.03	0.05	0.95**	−													
5 EL	0.03	0.11*	0.96**	0.87**	−												
6 MR	0.06	0.13*	0.94**	0.83**	0.85**	−											
7 CAMM	0.19**	–0.00	–0.04	–0.02	0.01	–0.07	−										
8 AFQ	−0.29**	0.01	0.02	0.02	0.00	0.06	−0.64**	−									
9 FC	−0.12*	0.02	0.01	0.01	–0.00	0.08	−0.58**	0.86**	−								
10 EA	−0.36**	0.00	0.02	0.02	0.01	0.03	−0.57**	0.93**		−							
11 WBSI	−0.14**	0.01	0.06	0.06	0.08	0.11**	−0.59**	0.59**	0.49**	0.57**	−						
12 Suppre.	–0.07	–0.01	0.06	0.04	0.05	0.09	−0.51**	0.54**	0.43**	0.52**	0.93**	−					
13 Intrus.	−0.13**	0.02	0.09	0.06	0.07	0.10	−0.55**	0.52**	0.45**	0.49**	0.95**	0.75**	−				
14 POS	–0.05	0.15*	0.53**	0.53**	0.57**	0.58**	0.03	–0.04	–0.07	–0.02	0.04	0.01	0.07	−			
15 NEG	−0.11**	0.13*	–0.07	–0.07	–0.08	–0.01	−0.48**	0.40**	0.40**	0.33**	0.35**	0.32**	0.32**	−0.15**	−		
16 BAL	0.04	0.01	0.42**	0.39**	0.42**	0.38**	0.34**	−0.30**	−0.31**	−0.24**	−0.21**	−0.21**	−0.17**	0.75**	−0.77**	−	
17 ANX-S	–0.09	−0.15**	−0.16**	−0.12*	−0.14**	−0.19**	−0.39**	0.31**	0.34**	0.24**	0.26**	0.23**	0.27**	−0.29**	0.37**	−0.48**	−
18 ANX-T	−0.18**	0.07	0.04	0.01	0.01	0.10	−0.60**	0.55**	0.50**	0.49**	0.48**	0.41**	0.47**	–0.01	0.62**	−0.42**	0.37**

*In bold significative relationships. ^∗^*p* < 0.05; ^∗∗^*p* < 0.01 3 ESCQ, TEI Total average scores; 4 PU, Perceiving and Understanding Emotions; 5 EL, Expressing and Labeling Emotions; 6, Managing and Regulating Emotions; 7 CAMM, DM Total Average; 8 AFQ, Cognitive Inflexibility Total Average; 9 FC, Fusion cognitive; 10 EA, Experiential avoidance; 11 WBSI, White bear suppression inventory; 12 Suppre., suppression of thoughts; 13 Intrus., Intrusion of thoughts; 14 POS, Positive Affect PANASN; 15 NEG, Negative Affect PANASN; 16 BAL, Balance (POS – NEG) PANASN; 17 ANX-S, Anxiety state STAI-C; and 18 ANX-T, Anxiety Trait STAI-C.*

Note that gender was related significantly with positive and negative affect of PANASN, anxiety state of STAI-C, and two subscales of ESCQ (expression and labeling emotions, and emotional managing and regulation). According to this finding, we checked whether there were significant outcomes with these criteria. In light of the observed gender differences and the theoretical considerations outlined in the introduction, we report all subsequent analyses separately by gender only for PANASN and Anxiety state. Apparently, on this occasion gender is a natural/cultural factor rather than a mediator. Hence, we used multiple regression analyses to examine associations between TEI and CAMM with these criteria controlling for just age.

Among boys, and after controlling for age, *Expression and labeling emotions* remained significantly associated with *positive affect* [*F*(5,155) = 14.42, *p* < 0.01; ß = 0.42, *p* < 0.001]. However, *DM*, CAMM (Spanish 8-items version) was associated with *negative affect* (PANASN) [*F*(5,155) = 11.92, *p* < 0.001; ß = −0.47, *p* < 0.001], but no measures of TEI (ESCQ) were related to negative affect.

Among girls, and after controlling for age, however, Age, DM, and TEI were related to *positive affect* of PANASN *affect* [*F*(5,149) = 25.69, *p* < 0.001]. *Emotional managing and regulation* (ß = 0.55, *p* < 0.001) and *dispositional mindfulness* (ß = 0.21, *p* < 0.01) remained significantly and positively associated with *positive affect.* However, *age* (ß = −0.16, *p* = 0.012) was negatively related to *positive affect.* Regarding *negative affect*, only *dispositional mindfulness* was significant [*F*(5,1149) = 8.88, *p* < 0.001; ß = −0.48, *p* < 0.001]. As with boys, and controlling for *age*, *anxiety state* remained negatively significant [*F*(5,149) = 9.26, *p* < 0.001] with *dispositional mindfulness* (ß = −0.45, *p* = 0.002) and *emotional managing and regulation* (ß = −0.33, *p* < 0.001). Reported multiple regression analyses that control for age were just significant among girls in negative affect, which may be explained because age is confused with academic achievement in this sample, given that students who repeat a grade tend to be older. However, we repeated the main analyses controlling simultaneously for age with the rest of the criteria and did not find essentially identical results.

Regarding AFQ-Y, *cognitive inflexibility*, we did not run further analyses beyond correlations due to the fact that it is well known the strong relationship existing between dispositional mindfulness and cognitive inflexibility (see Tay and Kuykendall, 2016). Our study found moderate and negative relationships (we recall that we recode CAMM items for a better interpretation) between *DM* and *fusion cognitive* (*r* = −0.58, *p* < 0.01) and *experiential avoidance* (*r* = −0.57, *p* < 0.01). However, TEI measures did not show significant relationships for these criteria.

All measures from White Bear Suppression Inventory (WBSI) were negatively and significantly (*p* < 0.001) related to *DM* (*r*_total_ = −0.59, *r*_suppression_ = 0.51, and *r*_intrusion_ = 0.55). However, just total scores of WBSI had significant correlations with *age* and *emotional managing and regulation.* Nonetheless, only *DM* was significant after multiple regression [*F*(3,313) = 55.78, ß = 0.57, *p* < 0.001].

Regarding *Anxiety trait*, while among boys just *DM* remained significant [*F*(4,156) = 63.93, *p* < 0.001, ß = *0.68, p* < *0.001*], girls obtained also *Emotional managing and regulation* (MR) [*F*(4,150) = 31.11, *p* < 0.001, ß*_*MR*_* = 0.24, *p* < 0.05, ß*_*DM*_* = −0.65, *p* < 0.001].

**FIGURE 1:**
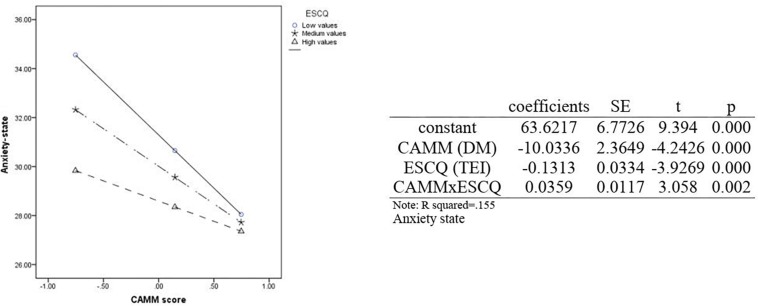
Moderation effect of TEI among DM and Anxiety state.

Finally, *Anxiety state* was significant with *emotional regulation* (MR) and *dispositional mindfulness* (DM) for both boys [*F*(4,155) = 9.09, *p* < 0.001, ß*_*MR*_* = −0.43, *p* < 0.01, ß*_*DM*_* = 0.41, *p* < 0.001] and girls [*F*(4,150) = 11.65, *p* < 0.001, ß*_*MR*_* = −0.33, *p* < 0.01, ß*_*DM*_* = −0.45, *p* < 0.001]. This is the only moderation relationship among CAMM, TEI, and a criterion (anxiety state) that was significant. Below, Figure 1 shows the moderation effect when CAMM (DM) is predicting anxiety estate, moderated by TEI (ESCQ total score).

## Discussion and Conclusion

Our main purpose was to study potential significant relations that both TEI and the DM may have with diverse criteria of the affective sphere, anxiety (trait and state), psychological inflexibility, and suppression and intrusion of thoughts. As it was noted earlier, we expected that TEI were more related to positive affect rather than with negative affect and suppression and intrusion of thoughts. But we also hypothesized a better prediction of DM (negatively) among negative emotional and cognitive criteria than TEI.

Regarding EI, our TEI measure is theoretically based on AEI theory (Mayer et al., 2016) and it was cross-culturally validated by Faria et al. (2006) and, besides, ESCQ has been reported to have good psychometric properties (Takšić et al., 2009). However, it should be considered as a TEI measure rather than an AEI one because TEI is measured using self-report tests and has been described as an “emotion-related dispositional trait which forms part of the personality domain” (Narula and Rugman, 2014, p. 1). Hence, ESCQ should have also common variance with the scales derived from similar constructs (convergent validity) of personality trait (see Faria et al., 2006). Furthermore, TEI factor has been reported as a distinct oblique within the Big Five space (Pérez-González and Sánchez-Ruiz, 2014).

With these caveats in mind, our TEI findings should be interpreted as tentative. Nevertheless, it is interesting how once again gender presented a key role in understanding how participants perceived themselves differently their emotional skills, especially children and adolescents (Windingstad et al., 2011). Among boys, we found that *expressing and labeling emotions* were positively and significantly related to positive affect. However, among girls, positive affect was combined, related to *emotional managing and regulating* and DM. We have already pointed how Teal et al. (2018) studied the influence of EI and DM in the well-being school programs in a sample of 294-male adolescents, where DM and TEI were linked to explain happiness. Despite using a different measure of TEI and positive criterion than these authors, we did not find this mediating relationship among boys, but we did among girls.

Male and female adolescents may use their emotional skills differently to develop a better perception of their positive affect. Although ESCQ is a self-reported measure, as TEI instruments are, it is also based on an AEI framework. In this same direction, others studies using AEI performance measures in male-and-female adolescents have pointed out how girls prefer to use emotional regulation abilities to improve their social functioning and positive states while male adolescents prefer to use them to achieve goals (Mestre et al., 2006; Lopes et al., 2012; Billings et al., 2014; Hawn et al., 2015; Fernandez-Berrocal and Extremera, 2016).

In our study, female participants that scored higher in DM also scored higher in the positive affect of PANASN. Some meta-analyses have reported a mediating effect of TEI in DM (Tomlinson et al., 2017; Miao et al., 2018), although gender was not considered as a factor of positive mental health. Regarding the possibility of female adolescents having better dispositional mindfulness for both social and self-functioning, further research including both factors (EI and DM) is needed. This is especially important if samples are heterogeneous (without academic filters), and more studies are necessary to examine gender as a mediator. In earlier stages of development, boys and girls have shown differences both in TEI studies (O’Connor and Little, 2003; Tett et al., 2005; Billings et al., 2014; Mikolajczak et al., 2014) and AEI measures (Allen et al., 2014; Fernandez-Berrocal and Extremera, 2016; Lopes et al., 2012; Mestre et al., 2006, 2017). These consistent outcomes involve an integrated intervention for developing positive outcomes among adolescents considering these gender differences. For example, Turanzas et al. (2018) have reported a combined treatment of DM and EI for gifted children based on second-generation of mindfulness-based on interventions (Van Gordon et al., 2015), where gifted children received mindful sessions and EI sessions with promising findings.

Another point of view and two questions also arise from our research: Does emotional intelligence predict the criteria of positive psychology better than dispositional mindfulness? Is DM a better predictor of outcome related to clinical psychology (anxiety, stress, negative emotions) than EI? Further research is needed to clarify this question but some antecedents from both EI models suggest a tentative-affirmative answer for the first question. From AEI research with children and adolescents, EI is related to a healthier psychological functioning (Rivers et al., 2012), a better resilience linked to emotional regulation ability (Mestre et al., 2017), a good understanding of complex emotional written (Barchard et al., 2013), a promoting adaptation (Davis et al., 2015), and more adaptive and positive emotional strategies (Peña-Sarrionandia et al., 2015). From TEI research with children and adolescents, individual differences in TEI appear to be a good predictor for social functioning: across the life span (Petrides et al., 2016), better academic performance (Petrides, 2016), social acceptation (Windingstad et al., 2011), and for personal functioning (Waugh et al., 2008; Laborde and Allen, 2016). However, with negative social functioning criteria (f.i., social misconduct), the findings have been indicated with the label “shreds of evidence are not as strong as we would like,” especially with AEI (Mavroveli et al., 2008; Fiori and Antonakis, 2011; Kong, 2014; Laborde and Allen, 2016). So, EI may be a stronger predictor for positive than negative adaptive criteria.

Regarding the second question, whether DM is a better predictor or not of outcomes related to clinic psychology (anxiety, stress, negative emotions) than EI. While our TEI measure was more related to positive outcomes, higher scores of DM were more related to lower scores of both negative emotional and cognitive criteria. Our findings matched with various studies about DM and positive and negative criteria [for children-and-adolescent studies (see Zhang and Wu, 2014; Roemer et al., 2015; Park and Dhandra, 2017b; Tomlinson et al., 2017)]. And female participants showed a combination of DM and EI effects for some criteria.

Due to some limitations (measures, sample sizes, and sample without EI and/or DM interventions among others), our findings should be interpreted tentatively. Further research may clarify if it is worth combining DM and EI designs in interventions with children and adolescents can combine DM and EI designs. If EI increases positive emotions and DM can slow-down negative emotions, then instructors could achieve wider outcomes in their interventions.

## Compliance With Ethical Standards

All procedures performed in studies involving human participants were in accordance with the ethical standards of the institutional and/or national research committee and with the 1964 Helsinki declaration and its later amendments or comparable ethical standards. We also followed the Spanish Law regarding Data Protection. According to article 13.1 of the Spanish Organic Law of Data Protection, the “data of persons over 14 years of age may be processed with their consent, except in those cases in which the Law requires the assistance of the holders of parental authority or guardianship. In the case of minors under 14 years of age, the consent of the parents or guardians will be required.”

## Informed Consent

Children and adolescents from this research participated voluntarily and they had to sign an informed consent according to the Spanish Organic Law of Data Protection. Written informed consent was obtained from the parents/legal guardians of all participants.

According to the Research and Ethical Committee of INDESS (Institute for University Research on Social and Sustainable Development, University of Cadiz, Spain), we had to follow following ethical recommendations: (a) all participants had to bring an informed consent from their parents, especially minors under 14 years old; (b) we had to inform and receive permission from AMPA —“Asociación de Padres y Madres del Centro Escolar” (every Spanish school has its own parents association), and (c) the study had to be approved by the Cadiz Education Office of the Andalusian Government. Accordingly, the study was approved by the AMPA and, the Cadiz Education Office of the Andalusian Government, and Ethics and Research Committee of INDESS.

## Ethics Statement

This study was carried out following the recommendations of the Scientific Integrity and Ethics at CSIC guidelines (Spanish Higher Council for Scientific Research). We also proceeded to gather the written informed consent from all participants, which was written under the Declaration of Helsinki. The protocol was also approved by the Ethical Research Committee of INDESS (University Institute of Research on Sustainable and Social Development, Universidad de Cádiz, Spain).

## Author Contributions

JM and VL-R designed the research and wrote and revised the whole process of the manuscript. JT, MG-G, and JG led the research and conducted the investigation. JC and JM made the statistical analyses. GT revised and contributed to the writing and revision of the manuscript.

## Conflict of Interest

The authors declare that the research was conducted in the absence of any commercial or financial relationships that could be construed as a potential conflict of interest.
